# Gene expression over the course of schizophrenia: from clinical high-risk for psychosis to chronic stages

**DOI:** 10.1038/s41537-019-0073-0

**Published:** 2019-03-28

**Authors:** Vanessa Kiyomi Ota, Patricia Natalia Moretti, Marcos Leite Santoro, Fernanda Talarico, Leticia Maria Spindola, Gabriela Xavier, Carolina Muniz Carvalho, Diogo Ferri Marques, Giovany Oliveira Costa, Renata Pellegrino, Simone de Jong, Quirino Cordeiro, Hakon Hakonarson, Gerome Breen, Cristiano Noto, Rodrigo Affonseca Bressan, Ary Gadelha, Jair de Jesus Mari, Sintia I. Belangero

**Affiliations:** 1 Disciplina de Genética, Departamento de Morfologia e Genética, Universidade Federal de São Paulo (UNIFESP), São Paulo, Brazil; 2 Laboratório Interdisciplinar de Neurociências Clínicas (LiNC), Departamento de Psiquiatria, Universidade Federal de São Paulo (UNIFESP), São Paulo, Brazil; 3 Departamento de Psiquiatria, Universidade Federal de São Paulo (UNIFESP), São Paulo, Brazil; 4 Faculdade de Medicina, Universidade de Brasília (UNB), Brasília, Brazil; 5 Center for Applied Genomics, Children’s Hospital of Philadelphia, Philadelphia, PA USA; 6 MRC Social Genetic and Developmental Psychiatry Centre, Institute of Psychiatry Psychology and Neuroscience, King’s College London, London, UK; 7 Departamento de Psiquiatria, Santa Casa School of Medical Sciences, São Paulo, Brazil

## Abstract

The study of patients with schizophrenia (SZ) at different clinical stages may help clarify what effects could be due to the disease itself, to the pharmacological treatment, or to the disease progression. We compared expression levels of targeted genes in blood from individuals in different stages of SZ: clinical high risk for psychosis (CHR), first episode of psychosis (FEP), and chronic SZ (CSZ). Then, we further verified whether single-nucleotide polymorphisms (SNPs) could be related to gene expression differences. We investigated 12 genes in 394 individuals (27 individuals with CHR, 70 antipsychotic-naive individuals with FEP, 157 CSZ patients, and 140 healthy controls (HCs)). For a subsample, genotype data were also available, and we extracted SNPs that were previously associated with the expression of selected genes in whole blood or brain tissue. We generated a mediation model in which a putative cause (SNP) is related to a presumed effect (disorder) via an intermediate variable (gene expression). *MBP* and *NDEL1* were upregulated in FEP compared to all other groups; *DGCR8* was downregulated in FEP compared to HC and CHR; *DGCR2* was downregulated in CSZ compared to FEP and HCs; *DISC1* was upregulated in schizophrenia compared to controls or FEP, possibly induced by the rs3738398 and rs10864693 genotypes, which were associated with *DISC1* expression; and *UFD1* was upregulated in CSZ and CHR compared to FEP and HC. Our results indicated changes in gene expression profiles throughout the different clinical stages of SZ, reinforcing the need for staging approaches to better capture SZ heterogeneity.

## Introduction

Schizophrenia (SZ) is a heterogenous disorder, with a wide array of clinical, functional, and cognitive outcomes. The different disease trajectories, in which a patient can present distinct clinical and biological features of disease progression, are a one major source of heterogeneity. Clinical staging models have been proposed, but relatively few studies compare biological measures in the distinct stages.^1^

Empirical operational criteria were developed to identify individuals with prodromal symptoms prior to the disease onset, which is the at-risk mental state (also called ultra-high risk, abbreviated CHR) that may convert into the first episode of psychosis (FEP) and then into chronic schizophrenia (CSZ).^2^ In individuals reaching these CHR criteria, the mean transition risk to a full-blown psychotic episode is 29.2% (95% confidence interval (CI) = 27.3–31.1%), within a mean follow-up of 31 months.^3^ However, considering that these CHR individuals are clinically heterogeneous and may reach different outcomes, including the remission of symptoms, the need exists for more predictive markers and an understanding of the biological mechanisms underlying the onset of psychosis.^4^ To allow for the identification of these markers, a better understanding of the biological changes in the different stages of schizophrenia is essential.

Considering that schizophrenia is a multifactorial disorder, both genetic variants and environmental factors are important for its etiology. Although the heritability of schizophrenia is very high (~80%), genetics still lack a major impact in clinical practice. Gene expression, the transcription of a gene’s DNA information into an RNA copy, is also influenced by a combination of environmental and genetic factors, such as expression quantitative trait loci (eQTLs), which are genomic loci that contribute to variation in expression levels. Schizophrenia risk loci have been noted as being enriched for eQTLs.^5^

Although many studies have investigated gene expression in the blood of patients with schizophrenia,^6^ most were performed in patients with a long time of treatment and disease. Our previous studies have shown that antipsychotics affect gene expression and DNA methylation,^7–9^ suggesting that gene expression may be influenced by the time of treatment and disease. Other studies have investigated prodromal or FEP patients, but no study has compared RNA expression between patients in different stages.

In this study, we investigated changes in the expression of 12 genes in different stages of schizophrenia: prior to disease onset (CHR), during the conversion to psychosis (FEP), during chronic schizophrenia (CSZ), and in healthy controls (HCs). Our objective is to find genes related to a prepsychotic stage (i.e., genes differentially expressed in CHR compared to other groups), to an acute psychotic stage (i.e., genes differentially expressed in FEP compared to other groups), to a long-term psychotic state, or following a long exposure to antipsychotics (i.e., genes differentially expressed in CSZ compared to the other groups). Moreover, for the differentially expressed genes, we selected single-nucleotide polymorphisms (SNPs) that were considered to be eQTLs to verify whether they are associated with schizophrenia or correlated to expression of their genes, thereby generating a mediation model in which a putative cause (SNP) is related to a presumed effect (disorder) via an intermediate variable (gene expression). In this way, we could verify whether the gene expression changes that we found are related to genomic variants or to other factors (e.g., environmental factors), and once such a relationship has been established, whether these genomic variants could be directly associated with schizophrenia or indirectly via gene expression.

## Results

In our comparison of the HC, CHR, FEP, and CSZ patients, we did not observe differences in sex, but as expected, a significant association with age was found (Table 1). Post hoc analyses showed that all groups were different from each other for age (adjusted *p* value < 0.05), with a lower age mean for CHR and a higher mean for CSZ.

**Table 1:** Demographic characteristics and gene expression of the cohorts

	HCs	CHR	FEP	SZ	*P* value
Gender (%)	M: 73 (52.1)	M: 17 (63)	M: 42 (60)	M: 100 (63.7)	0.226^a^
	F: 67 (47.9)	F: 10 (37)	F: 28 (40)	F: 57 (36.3)	
Age in years (SD)	33.51 (11.94)	18.00 (3.27)	25.86 (7.49)	38.65 (10.82)	<0.001^b^
*COMT* (SD)^c^	5.62 (0.31)	5.60 (0.34)	5.74 (0.33)	5.55 (0.37)	0.112^d^
*TNF* (SD)^c^	7.01 (0.45)	7.19 (0.35)	7.18 (0.51)	6.85 (0.50)	0.372^d^
*AKT1* (SD)^c^	4.32 (0.27)	4.31 (0.28)	4.33 (0.34)	4.30 (0.30)	1.000^d^
*MBP* (SD)^c^	**8.79** (**0.63)**	**9.29** (**0.92)**	**8.42** (**0.76)**	**8.93** (**0.54)**	**<0.001** ^d^
*DGCR8* (SD)^c^	**7.05** (**0.43)**	**7.00** (**0.41)**	**7.30** (**0.53)**	**7.17** (**0.47)**	**0.031** ^d^
*DICER1* (SD)^c^	5.52 (0.37)	5.53 (0.43)	5.49 (0.41)	5.42 (0.39)	1.000^d^
*DROSHA* (SD)^c^	6.60 (0.42)	6.47 (0.60)	6.85 (0.54)	6.65 (0.44)	0.056^d^
*DGCR2* (SD)^c^	**4.72** (**0.42)**	**4.60** (**0.40)**	**4.54** (**0.35)**	**4.94** (**0.41)**	**<0.001** ^d^
*UFD1* (SD)^c^	**7.29** (**0.51)**	**7.07** (**0.40)**	**7.51** (**0.45)**	**6.98** (**0.47)**	**<0.001** ^d^
*DISC1* (SD)^c^	**8.66** (**0.69)**	**8.15** (**0.73)**	**8.62** (**0.68)**	**8.37** (**0.88)**	**0.005** ^d^
*NDEL1* (SD)^c^	**4.07** (**0.32)**	**4.12** (**0.41)**	**3.79** (**0.40)**	**4.06** (**0.38)**	**<0.001** ^d^
*PAFAH1B1* (SD)^c^	4.74 (0.25)	4.71 (0.30)	4.84 (0.31)	4.70 (0.29)	0.133^d^

*HC* healthy controls, *CHR* clinical high risk, *FEP* first episode of psychosis, *CSZ* chronic schizophrenia, *M* male, *F* female

Bold values mean that the *p*-value are statistically significant considering a alpha <0.05

^a^*P-* value from Pearson’s chi-squared test comparing the four groups

^b^*P* value from analysis of variance (ANOVA) test comparing the four groups

^c^Mean presented in ΔCrt which is negatively correlated with gene expression

^d^*P* value from general linear model (GLM) comparing the four groups. ΔCrt was inserted as dependent variable, group and gender as fixed factors, and age as covariate. Bonferroni correction for 12 comparisons was applied

*MBP*, *DGCR8*, *DGCR2*, *UFD1*, *DISC1*, and *NDEL1* were differentially expressed among groups after Bonferroni correction for 12 comparisons, controlling for age and sex (Table 1).

**Fig. 1:**
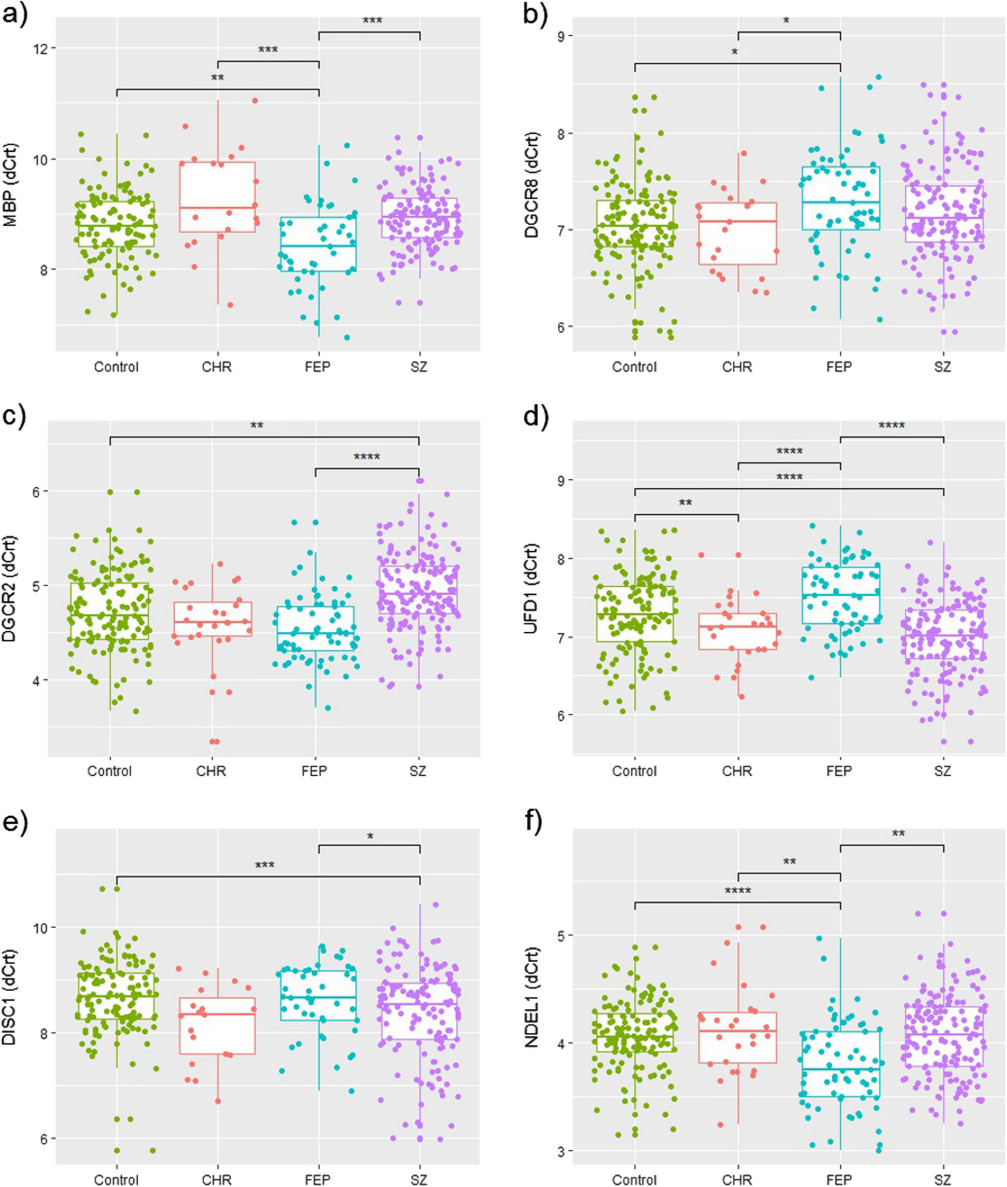
Boxplots representing ΔCrt (which is negatively correlated with gene expression) of each differentially regulated gene among healthy controls (HCs, *N* = 140), clinical high risk (CHR, *N* = 27) individuals, antipsychotic-naive first episode of psychosis (FEP, *N* = 70) patients, and chronic schizophrenia (CSZ, *N* = 157) patients. **a** *MBP* gene (Myelin Basic Protein); **b** *DGCR8* gene (DiGeorge Syndrome Critical Region Gene 8, Microprocessor Complex Subunit); **c** *DGCR2* gene (DiGeorge Syndrome Critical Region Gene 2); **d** *UFD1* gene (Ubiquitin Recognition Factor In ER Associated Degradation 1); **e** *DISC1* gene (Disrupted in Schizophrenia 1 Scaffold Protein); **f** *NDEL1* gene (NudE Neurodevelopment Protein 1 Like 1). Boxplot center lines = median; lower bound = 25% quantile; upper bound = 75% quantile; lower whisker = smallest observation greater than or equal to lower hinge − 1.5 × interquartile range (IQR); upper whisker = largest observation less than or equal to upper hinge + 1.5 × IQR; *post hoc *p* < 0.05; **post hoc *p* < 0.01; ***post hoc *p* < 0.001; ****post hoc *p* < 0.0001

Bonferroni post hoc analyses showed that *MBP* and *NDEL1* were upregulated in FEP compared to all the other groups; *DGCR8* was downregulated in FEP compared to the HCs and CHR; *DGCR2* was downregulated in CSZ compared to the HCs and FEP; *UFD1* was upregulated in CSZ compared to the HCs and FEP and in CHR compared to HCs and FEP; and *DISC1* was upregulated in CSZ compared to the controls and FEP (Fig. 1).

SNP association was performed on a subsample of 132 HCs, 60 FEP patients, and 108 CSZ individuals. Among the differentially expressed genes, only *DISC1* (278 SNPs) and *DGCR8* (17 SNPs) presented eQTLs in whole blood for our available genotyped data, and *NDEL1* (2 SNPs), *DGCR2* (214 SNPs), and *MBP* (2 SNPs) presented eQTLs in brain tissue. None of these SNPs were significantly associated with SZ (considering FEP and CSZ individuals) independently (*p* > 0.05). Considering only eQTLs in whole blood, we found a significant association between *DISC1* ΔCrt and rs3738398 (*p* = 0.013) and rs10864693 (*p* = 0.045). For both SNPs, we observed the same pattern as reported in GTEx (rs3738398: GG<GC<CC; rs10864693: TT<TG <GG), considering that ΔCrt is negatively correlated with gene expression (Supplementary Figure S1).

Since we did not find an association between the SNP and the disorder, we analyzed whether these SNPs were associated with SZ via an indirect effect of gene expression (Supplementary Figure S2). For this analysis, we tested rs3738398 and rs10864693 because they were related to *DISC1* expression. Indeed, when we selected only CSZ patients and HCs, excluding FEP patients, we observed an indirect mediating effect for each SNP on schizophrenia diagnosis via *DISC1* expression (rs3738398: effect = 0.135, 95% bootstrap CI = 0.009–0.346; rs10864693: effect = 0.120, 95% bootstrap CI = 0.004–0.313). In terms of proportion of mediated effect, we have for model rs3738398 ->*DISC1* expression ->disorder 91% is mediated by the *DISC1* expression and for rs10864693 ->*DISC1* expression ->disorder model 70.61% of the effect is mediated by *DISC1* expression. No significant direct or indirect effect was found when we compared FEP patients and HCs or cases (considering FEP and CSZ individuals) and HCs.

## Discussion

The study of patients with schizophrenia at different clinical stages, from at-risk mental states to chronicity, may help clarify what effects could be due to the disease itself, to the pharmacological treatment, or to the disease progression. We could hypothesize that genes with a distinct expression in FEP might be related to psychosis and stress; those with a unique expression in CSZ could reflect changes related to antipsychotic treatment; and those with a different pattern could be related to illness progression or interaction with antipsychotics. Additionally, we cannot rule out an influence of other factors, such as eQTLs, even though we investigated those inferred by GTEx, as described below, or other external factors not measured, cited below in the limitations section. We found six genes differentially expressed among the different stages of schizophrenia. First, we found genes associated with an acute psychotic stage (*MBP* and *NDEL1*), as these genes were upregulated in FEP compared to all other groups. This could reflect changes related to stress and the onset of psychosis, which were attenuated in the other SZ stages (CHR and CSZ) or in the absence of the disorder (HC). Second, although *DGCR8* was also differentially expressed in FEP compared to the HCs and CHR, after individuals were exposed to antipsychotics and 3 years of the disorder, these levels seem to increase slightly in CSZs, and in an intermediate level between FEP and HCs/CHRs. Therefore, this gene may be influenced by both psychosis and antipsychotic treatment. Third, *DGCR2* might reflect antipsychotic treatment or illness time, considering that it is downregulated in CSZ compared to antipsychotic-naive FEP patients and HCs. Fourth, *DISC1* expression was associated with rs3738398 and rs10864693 genotypes, which may underlie the upregulation in CSZ compared to controls or FEP. Interestingly, these SNPs were not directly associated with schizophrenia; however, via a mediation model, they were indirectly associated with the disorder through *DISC1* expression. Finally, *UFD1* expression may be associated with antipsychotic treatment, since it is upregulated in CSZ and CHR (5/27 CHR individuals were treated) compared to antipsychotic-naive FEP and HCs. A brief description of each gene is provided in Supplementary Table S1.

We previously reported the *MBP* and *NDEL1* upregulation in FEP and FEP with mania compared to controls^10,11^ and in FEP compared to CHR^1^; however, here we show that these changes are specific to this acute psychotic stage and are not found in later (CSZ) stages. Previous studies in postmortem brain revealed decreased *MBP* mRNA and protein levels in schizophrenia patients.^12–15^ However, *Mbp* expression was shown to be modulated by antipsychotic treatment in mouse^16^ and in an independent study, it was found to be upregulated in the blood of antipsychotic-naive schizophrenia patients.^17^

*NDEL1* downregulation was reported in the hippocampus and blood of patients with schizophrenia.^18^ Notably, NDEL1 is a known DISC1 interactor, which has been associated with schizophrenia and other major mental disorders.^19^ Here, we found that two SNPs regulated *DISC1* expression, corroborating their role as eQTLs for whole blood found in GTEx, and even more interesting, these SNPs were associated with SZ in an indirect way, via *DISC1* expression. *DISC1* was upregulated in CSZ compared to FEP and HCs; thus, we hypothesize that differences in its expression reflect both genetic variants and environmental factors (e.g., antipsychotic treatment or duration of illness), since we did not find an association when FEP patients and HCs were compared. Indeed, some antipsychotics seem to increase *DISC1* expression.^20^ In an independent study, Kumarasinghe et al.^17^ found *DISC1* was upregulated in peripheral blood mononuclear cells of treatment-naive schizophrenia patients and persisted after 6 weeks of antipsychotic drug treatment. Moreover, Nakata et al.^21^ reported increased expression of specific isoforms of DISC1 in the hippocampus in patients with schizophrenia.

*DGCR2*, *DGCR8*, and *UFD1* are deleted in individuals with 22q11.2 deletion syndrome (22q11DS), which occurs in individuals who have increased susceptibility for psychiatric disorders. DGCR8 protein participates in the processing of microRNA molecules (miRNAs), which are noncoding RNAs that are typically 22 nucleotides long and regulate gene expression. *Dgcr8* deficiency in mice leads to downregulation of a subset of mature miRNAs, smaller dendritic spines, a simpler dendritic tree, alterations in synaptic properties, and cognitive and behavioral deficits.^22,23^ We found a *DGCR8* downregulation in FEP compared to HCs and CHR, which may corroborate the association between 22q11 deletion and schizophrenia. However, Beveridge et al.^24^ observed that *DGCR8* was upregulated in postmortem dorsolateral prefrontal cortex and superior temporal gyrus of subjects with schizophrenia. Thus, we could hypothesize that *DGCR8* is downregulated in early psychotic stages and may be affected by antipsychotic treatment, which could be increasing its expression in an attempt to return to normal levels, since we did not observed gene expression differences between CSZ and FEP. We could also infer that *DGCR8* might be more related to psychotic symptoms, which are more pronounced during the FEP stage.

Similar to *DGCR8*, *DGCR2* was found to be upregulated in the dorsolateral prefrontal cortex of schizophrenia patients.^25^ The same study also observed that antipsychotic drugs elevated *Dgcr2* expression in rats. In our study, we observed *DGCR2* downregulation in the blood of CSZ patients, which could be reflecting antipsychotic treatment effects in blood, since this was not found in antipsychotic-naive FEP patients. Our results for *UFD1*, previously known as *UFD1L* and previously reported as being upregulated in CSZ and CHR,^1,26^ showed that this alteration is also observed when comparing CSZ and FEP. Considering that five of the CHR were not antipsychotic naive, these *UFD1* alterations could be related to antipsychotic treatment, since both CHR and CSZ presented an upregulation in this gene.

Our major limitation is the small sample size, particularly for the CHR group; however, we were still capable of finding differences in gene expression depending on the stage of the disorder and the antipsychotic treatment. Moreover, considering that gene expression is tissue and time specific, we cannot extrapolate our findings in whole blood to brain tissues, although whole blood markers can be better for prognosis, and brain markers can be useful for understanding the pathophysiology of the disorder. Therefore, our findings might not be related to schizophrenia pathogenesis. Despite this fact, a comparison of gene expression among different stages of schizophrenia is not possible in brain tissues since collecting these samples from CHR and antipsychotic-naive FEP is very difficult. We also cannot rule out that the differences that we found were not due to other external factors, such as smoking, other medications, diet, and trauma, considering that they can potentially influence gene expression, although we tested for some samples (Supplementary Notes). Finally, in an ideal study, we should have investigated different stages of SZ longitudinally in the same people, instead of comparing different individuals among stages; however, very few studies have evaluated gene expression at different SZ stages. Some other limitations must be considered in terms of the mediation analysis results. We only adjusted for age (in both models) and, therefore, there are different unmeasured potential confounder factors that might explain indirect effects or even narrowing our possibility of infer causality in both models. Regarding the latter, the modern literature consider four main potential unmeasured confounders, where control must be made for (a) exposure–outcome confounding, (b) mediator–outcome confounding, (c) exposure–mediator confounding, and (d) there should be no mediator–outcome confounder that is itself affected by the exposure (for a review see ref. ^27^). Moreover, it is possible that the genotypes and *DISC1* expression will interact one with each other.

To our advantage, a need exists to account for antipsychotic effects and genetic variation in gene expression studies, which was investigated in our study and, interestingly, we found that two SNPs were associated indirectly with schizophrenia via gene expression. Our findings could be useful for creating a biological signature that is able to predict a psychotic event or help to find adequate treatment and decrease the illness time without a treatment. In this sense, a better characterization of each stage of schizophrenia is essential to improve the understanding of the pathogenesis of the disorder.

## Methods

### Subjects

A total of 394 subjects (27 CHR individuals, 70 antipsychotic-naive FEP patients, 157 CSZ subjects, and 140 HCs) were selected for gene expression analyses. Although we had already investigated these cohorts in previous studies separately,^1,10,11,26^ we had never compared these four cohorts among each other, representing a comparison among different stages of schizophrenia. Genotyping data were available for 132 HCs and 168 patients (60 FEP and 108 CSZ). All participants or their caregivers signed a written informed consent form approved by the Research Ethics Committee of Universidade Federal de São Paulo (UNIFESP).

CHR individuals were help-seeking individuals or subjects referred by primary and secondary care services recruited from the “Program of Recognition and Intervention in subjects At-Risk Mental States” (PRISMA). They were from 14 to 26 years of age and were included if CHR status was confirmed using the Comprehensive Assessment of At-Risk Mental States (CAARMS). Of the 27 CHR individuals, 5 were receiving antipsychotics at the time of blood collection, and none had converted to schizophrenia. FEP individuals were inpatients from 15 42 years of age who were recruited from the “Center for Integrated Mental Health of Santa Casa of São Paulo” (CAISM) and were interviewed by psychiatrists according to DSM-IV (Diagnostic and Statistical Manual of Mental Disorders, 4th Edition) criteria with Structured Clinical Interview for DSM Disorders (SCID-I). They were only included if they were antipsychotic naive and with diagnoses of schizophrenia, schizophreniform disorder, brief psychotic disorder, and psychotic disorder not otherwise specified. The CSZ group comprised chronic outpatients with at least 3 years of the disorder diagnosis according to DSM-IV criteria (SCID-I). They were 19–71 years old and recruited from the “Schizophrenia Program” (PROESQ) or CAISM. The HCs answered SCID-I and were included if they did not meet criteria for any Axis I DSM-IV mental disorder and if they had no family history of psychotic disorders in first-degree relatives.

### Gene expression and genotyping

Blood was collected in EDTA and PAXgene RNA tubes (PreAnalytiX, Hombrechtikon, Switzerland), and the DNA and RNA were isolated, respectively, with Gentra Puregene (Qiagen, Germantown, MD, USA) and PAXgene Blood RNA Kit (Qiagen) according to the manufacturer’s instructions.

For the gene expression analyses, approximately 400 ng of each RNA sample was reverse-transcribed using a High-Capacity cDNA Reverse Transcription Kit (Thermo Scientific, USA), and the complementary DNA (cDNA) was mixed with TaqMan Universal PCR Master Mix (Thermo Scientific), loaded on TaqMan Low-Density Array (TLDA) microfluidic cards (Thermo Scientific), which we used on a ViiA 7 Real-Time PCR System (Thermo Scientific). Gene expression was quantified using the relative threshold method (Crt) with the geometric mean (GM) between *ACTB* and *GAPDH* genes as endogenous control. Delta Crt values (ΔCrt = Crt_target gene_ − (Crt_GM_) were calculated for each sample and included in the PASW Statistics (version 18.0, SPSS, Chicago, IL, USA) data set.

Genes were selected based on their previous association with schizophrenia or psychotic disorders, with a focus on genes involved in neurodevelopment, myelination, neuroplasticity, neurotransmission, and miRNA biosynthesis. Selected target genes included *COMT* (Catechol-O-Methyltransferase), *TNF* (Tumor Necrosis Factor), *AKT1* (AKT Serine/Threonine Kinase 1), *MBP* (Myelin Basic Protein), *DGCR8* (DGCR8, Microprocessor Complex Subunit), *DICER1* (Dicer 1, Ribonuclease III), *DROSHA* (Drosha Ribonuclease III), *DGCR2* (DiGeorge Syndrome Critical Region Gene 2), *UFD1* (Ubiquitin Recognition Factor In ER Associated Degradation 1), *DISC1* (Disrupted in Schizophrenia 1 Scaffold Protein), *NDEL1* (NudE Neurodevelopment Protein 1 Like 1), and *PAFAH1B1* (Platelet Activating Factor Acetylhydrolase 1b Regulatory Subunit 1).

For a subsample of 132 HCs, 60 FEP, patients and 108 schizophrenia individuals, genotype data were available and were generated from HumanOmniExpress BeadChips (Illumina, USA) and PsychChip (Illumina, USA). Quality control and genomic imputation were performed according to previous studies.^28,29^ We extracted the genotypes from SNPs that were considered to be eQTLs from whole blood or brain tissue of the differentially expressed genes in our analysis according to GTEx (www.gtexportal.org^30^).

### Statistics

First, outliers (those lying outside 2.2× IQR (interquartile range)) were removed. A general linear model (GLM) was used to associate gene expression levels and groups, with the ΔCrt as the dependent variable, group and sex as fixed factors, and age as the covariate. Bonferroni correction for 12 comparisons was applied, with an adjusted *p* < 0.05 considered as significant.

Association between genotypes and schizophrenia or gene expression was performed via Plink2 (https://www.cog-genomics.org/plink/2.0/) using max(T) for 10,000 permutations.

We applied a mediation model to associate a putative cause (SNP) to a presumed effect (having a schizophrenia diagnosis) via an intermediate variable (gene expression). For this analysis, the PROCESS version 2.16 for SPSS was performed, considering 10,000 bootstrap samples and age as a covariate. Significance of the indirect effects was tested based on bootstrapping resampling method (bias corrected bootstrap confidence intervals available in the PROCESS version 2.16 for SPSS), which works under ordinary least square estimator. Simulation research shows that bootstrapping is more powerful than the traditional Sobel test and the causal steps approach to testing intervening variable effects;^31,32^ under bootstrapping approach, the significance of the indirect effect is obtained if the confidence interval does not contain zero. Moreover, in terms of effect size we computed the proportion of the effect that is mediated, or the indirect effect divided by the total effect (whereas the total effect = direct + indirect effect). As described in ref., ^33^ this effect size might be informative, especially when c’ is not statistically significant

### Reporting Summary

Further information on experimental design is available in the Nature Research Reporting Summary linked to this article.

## Supplementary information

Supplemental Material

Reporting Summary

## Data Availability

The datasets generated during and/or analyzed during the current study are available from the corresponding author on reasonable request.

## Supplementary information

**Supplementary Information** accompanies the paper on the *npj Schizophrenia* website (10.1038/s41537-019-0073-0).
