# Supplementary material for: Gene expression over the course of schizophrenia: from clinical high-risk for psychosis to chronic stages
Source: NPJ Schizophr. 2019 Mar 28;5:5. doi: 10.1038/s41537-019-0073-0 (PMC6438978; doi:10.1038/s41537-019-0073-0)
Supplement: Supplementary file 1 — Supplemental Material [file 41537_2019_73_MOESM1_ESM.pdf]

Table S1: Information about the candidate genes selected for this study (biological system, official full name, function and citation of previous studies).

Biological System	Gene Symbol	Official Full Name	Function	References
**Neurodevelopment**	*DISC1*	*disrupted in schizophrenia 1*	Protein involved in neurite outgrowth and cortical development	Gene originally discovered at the breakpoint of a balanced translocation in families with high rates of schizophrenia (Sachs et al., 2005). Reduced *DISC1* levels in blood of patients with schizophrenia (Rampino et al., 2014). Antipsychotics seem to modify *DISC1* expression in frontal cortex of mice (Chiba et al., 2006).
**Neurodevelopment**	*NDEL1*	*NudE Neurodevelopment Protein 1 Like 1*	Protein involved in multiple processes including cytoskeletal organization, cell signaling and neuron migration, outgrowth and maintenance	NDEL1 protein interacts with DISC1 (Hayashi et al., 2010) and LIS1 (Lissencephaly 1) (Bradshaw and Hayashi, 2017). *NDEL1* expression levels are reduced in the dorsolateral prefrontal cortex, hippocampus and blood of schizophrenia patients (Lipska et al., 2006; Rampino et al., 2014).
**Neurodevelopment**	*PAFAH1B1*	*Platelet Activating Factor Acetylhydrolase 1b Regulatory Subunit 1*	Protein that interacts with microtubules and regulates the activity of a variety of proteins that are involved in their function	Haploinsufficiency of *PAFAH1B1* gene causes the cerebral cortical malformation lissencephaly. Its protein, LIS1 is a binding partner of NDEL1 (Bradshaw and Hayashi, 2017). Reduced mRNA levels were observed in the dorsolateral prefrontal cortex and hippocampus of patients with schizophrenia (Lipska et al., 2006).
**Neurodevelopment**	*AKT1*	*AKT Serine/Threonine Kinase 1*	Serine/threonine-protein kinase that regulates many processes including metabolism and proliferation; and, in central nervous system, is involved in	AKT1 was shown to be involved in the dopaminergic signaling (Beaulieu et al., 2009). Decreased AKT1 protein and mRNA levels were found in postmortem brain tissue and lymphocyte-derived cells of individuals with schizophrenia and

			neurodevelopment, synaptic plasticity and protein synthesis	bipolar disorder(Emamian et al., 2004; Thiselton et al., 2008; van Beveren et al., 2012a). In antipsychotic-naive schizophrenia patients, *AKT1* was elevated in blood; and antipsychotic pharmacotherapy seemed to compensate it (Kumarasinghe et al., 2013).
**Neurodevelopment**	*DGCR2*	*DiGeorge Syndrome Critical Region Gene 2*	Activity-dependent adhesion protein that has been suggested to regulate critical steps of early corticogenesis	*DGCR2* is located at chromosome 22q11.2, a region that is associated with schizophrenia and other neurodevelopmental disorders. DGCR2 common and rare variations are associated with schizophrenia (Shifman et al., 2006; Xu et al., 2011). Its expression is increased in dorsolateral prefrontal cortex in patients with schizophrenia and in rats under treatment with antipsychotic drugs when compared with non-treated rats (Shifman et al., 2006).
Myelination	*MBP*	*Myelin Basic Protein*	Protein that is a major constituent of the myelin sheath of oligodendrocytes and Schwann cells in the nervous system	MBP mRNA and protein levels are decreased in anterior temporal lobe, visual cortex and prefrontal cortex tissues from schizophrenia patients (Martins-de-Souza et al., 2009; Matthews et al., 2012; Tkachev et al., 2003)
**miRNA pathway**	*DGCR8*	*DiGeorge Syndrome Critical Region Gene 8, Microprocessor Complex Subuni*	Subunit of the microprocessor complex which mediates the biogenesis of microRNAs	Single-nucleotide polymorphisms in *DGCR8*, located at chromosome 22q11.2, were associated to schizophrenia in Chinese population (Zhou et al., 2013). *DGCR8* mRNA is upregulated in superior temporal gyrus and dorsolateral prefrontal cortex of schizophrenia patients (Beveridge et al., 2010)
**miRNA pathway**	*DICER1*	*Dicer 1, Ribonuclease III*	Protein that cleaves precursor RNA molecules to produce miRNA	*DICER1* single-nucleotide polymorphisms and copy-number variations are associated to schizophrenia

				(Xu et al., 2008; Zhou et al., 2013). *DICER1* levels are increased in the dorsolateral prefrontal cortex and lymphoblastoid cell lines of schizophrenia cases (Beveridge et al., 2010; Sanders et al., 2013; Santarelli et al., 2011). Valproic acid, a mood stabilizer used to treat bipolar disorder, induces DICER degradation (Zhang et al., 2013).
**miRNA pathway**	*DROSHA*	*Drosha Ribonuclease III*	Ribonuclease that catalyzes the initial processing step of miRNA synthesis	*DROSHA* mRNA levels are upregulated in the dorsolateral prefrontal cortex of patients with schizophrenia (Beveridge et al., 2010)
**Neurotransmission**	*COMT*	*catechol-O-methyltransferase*	It catalyzes the transfer of a methyl group from S-adenosylmethionine to catecholamines	Gene located at chromosome 22q11.2, *COMT* single-nucleotide polymorphisms and copy-number variations are associated to schizophrenia (Goes et al., 2015). Promoter hypomethylation-associated overexpression of *COMT* in the left frontal lobe of patients with schizophrenia and bipolar disorder (Abdolmaleky et al., 2006). Antipsychotics altered *Comt* expression in rats frontal cortex (Fatemi et al., 2012). Blood *COMT* expression levels seem to be associated with psychosis-proneness (Grant et al., 2014), schizophrenia and response to antipsychotics (Li et al., 2018).
**Protein degradation**	*UFD1*	*Ubiquitin Recognition Factor In ER Associated Degradation 1*	Protein within a complex that is necessary for the degradation of ubiquitinated proteins	*UFD1* is located at chromosome 22q11.2 and single nucleotide polymorphisms within this gene were associated with schizophrenia or its phenotypes (De Luca et al., 2001; Ota et al., 2010; Ota et al., 2013; Xie et al., 2008). Patients with 22q11.2 deletion syndrome show reduced expression of

				this gene in Peripheral Blood Mononuclear Cells (van Beveren et al., 2012b). Mouse models for this syndrome also show diminished expression of *UFD1* in developing and adult brain (Meechan et al., 2006).
**Inflammation**	*TNF*	*tumor necrosis factor*	A multifunctional proinflammatory cytokine involved in the regulation of a wide spectrum of biological processes including cell proliferation, differentiation and apoptosis	TNF protein and mRNA levels are altered in blood and prefrontal cortex of patients with schizophrenia (Freudenreich et al., 2010; Liu et al., 2010; Miller et al., 2011; Pandey et al., 2018). Antipsychotics seem to modify *TNF* mRNA expression in adipocytes and TNF plasma levels (Pollmacher et al., 1996; Sarvari et al., 2014)

References

Abdolmaleky, H.M., Cheng, K.H., Faraone, S.V., Wilcox, M., Glatt, S.J., Gao, F., Smith, C.L., Shafa, R., Aeali, B., Carnevale, J., Pan, H., Papageorgis, P., Ponte, J.F., Sivaraman, V., Tsuang, M.T., Thiagalingam, S., 2006. Hypomethylation of MB-COMT promoter is a major risk factor for schizophrenia and bipolar disorder. Hum Mol Genet 15(21), 3132-3145.

Beaulieu, J.M., Gainetdinov, R.R., Caron, M.G., 2009. Akt/GSK3 signaling in the action of psychotropic drugs. Annu Rev Pharmacol Toxicol 49, 327-347.

Beveridge, N.J., Gardiner, E., Carroll, A.P., Tooney, P.A., Cairns, M.J., 2010. Schizophrenia is associated with an increase in cortical microRNA biogenesis. Mol Psychiatry 15(12), 1176-1189.

Bradshaw, N.J., Hayashi, M.A., 2017. NDE1 and NDEL1 from genes to (mal)functions: parallel but distinct roles impacting on neurodevelopmental disorders and psychiatric illness. Cell Mol Life Sci 74(7), 1191-1210.

Chiba, S., Hashimoto, R., Hattori, S., Yohda, M., Lipska, B., Weinberger, D.R., Kunugi, H., 2006. Effect of antipsychotic drugs on DISC1 and dysbindin expression in mouse frontal cortex and hippocampus. J Neural Transm (Vienna) 113(9), 1337-1346.

De Luca, A., Pasini, A., Amati, F., Botta, A., Spalletta, G., Alimenti, S., Caccamo, F., Conti, E., Trakalo, J., Macciardi, F., Dallapiccola, B., Novelli, G., 2001. Association study of a promoter polymorphism of UFD1L gene with schizophrenia. Am J Med Genet 105(6), 529-533.

Emamian, E.S., Hall, D., Birnbaum, M.J., Karayiorgou, M., Gogos, J.A., 2004. Convergent evidence for impaired AKT1-GSK3beta signaling in schizophrenia. Nat Genet 36(2), 131-137.

Fatemi, S.H., Folsom, T.D., Reutiman, T.J., Novak, J., Engel, R.H., 2012. Comparative gene expression study of the chronic exposure to clozapine and haloperidol in rat frontal cortex. Schizophr Res 134(2-3), 211-218.

Freudenreich, O., Brockman, M.A., Henderson, D.C., Evins, A.E., Fan, X., Walsh, J.P., Goff, D.C., 2010. Analysis of peripheral immune activation in schizophrenia using quantitative reverse-transcription polymerase chain reaction (RT-PCR). Psychiatry Res 176(2-3), 99-102.

Goes, F.S., McGrath, J., Avramopoulos, D., Wolyniec, P., Pirooznia, M., Ruczinski, I., Nestadt, G., Kenny, E.E., Vacic, V., Peters, I., Lencz, T., Darvasi, A., Mulle, J.G., Warren, S.T., Pulver, A.E., 2015. Genome-wide association study of schizophrenia in Ashkenazi Jews. Am J Med Genet B Neuropsychiatr Genet 168(8), 649-659.

Grant, P., Gabriel, F., Kuepper, Y., Wielpuetz, C., Hennig, J., 2014. Psychosis-proneness correlates with expression levels of dopaminergic genes. Eur Psychiatry 29(5), 304-306.

Hayashi, M.A., Guerreiro, J.R., Charych, E., Kamiya, A., Barbosa, R.L., Machado, M.F., Campeiro, J.D., Oliveira, V., Sawa, A., Camargo, A.C., Brandon, N.J., 2010. Assessing the role of endooligopeptidase activity of Ndel1 (nuclear-distribution gene E homolog like-1) in neurite outgrowth. Mol Cell Neurosci 44(4), 353-361.

Kumarasinghe, N., Beveridge, N.J., Gardiner, E., Scott, R.J., Yasawardene, S., Perera, A., Mendis, J., Suriyakumara, K., Schall, U., Tooney, P.A., 2013. Gene expression profiling in treatment-naive schizophrenia patients identifies abnormalities in biological pathways involving AKT1 that are corrected by antipsychotic medication. Int J Neuropsychopharmacol 16(7), 1483-1503.

Li, Z., He, Y., Han, H., Zhou, Y., Ma, X., Wang, D., Zhou, J., Ren, H., Yuan, L., Tang, J., Zong, X., Hu, M., Chen, X., 2018. COMT, 5-HTR2A, and SLC6A4 mRNA Expressions in First-Episode Antipsychotic-Naive Schizophrenia and Association With Treatment Outcomes. Front Psychiatry 9, 577.

Lipska, B.K., Peters, T., Hyde, T.M., Halim, N., Horowitz, C., Mitkus, S., Weickert, C.S., Matsumoto, M., Sawa, A., Straub, R.E., Vakkalanka, R., Herman, M.M., Weinberger, D.R., Kleinman, J.E., 2006. Expression of DISC1 binding partners is reduced in schizophrenia and associated with DISC1 SNPs. Hum Mol Genet 15(8), 1245-1258.

Liu, L., Jia, F., Yuan, G., Chen, Z., Yao, J., Li, H., Fang, C., 2010. Tyrosine hydroxylase, interleukin-1beta and tumor necrosis factor-alpha are overexpressed in peripheral blood mononuclear cells from schizophrenia patients as determined by semi-quantitative analysis. Psychiatry Res 176(1), 1-7.

Martins-de-Souza, D., Gattaz, W.F., Schmitt, A., Rewerts, C., Marangoni, S., Novello, J.C., Maccarrone, G., Turck, C.W., Dias-Neto, E., 2009. Alterations in oligodendrocyte proteins, calcium homeostasis and new potential markers in schizophrenia anterior temporal lobe are revealed by shotgun proteome analysis. J Neural Transm (Vienna) 116(3), 275-289.

Matthews, P.R., Eastwood, S.L., Harrison, P.J., 2012. Reduced myelin basic protein and actin-related gene expression in visual cortex in schizophrenia. PLoS One 7(6), e38211.

Meechan, D.W., Maynard, T.M., Wu, Y., Gopalakrishna, D., Lieberman, J.A., LaMantia, A.S., 2006. Gene dosage in the developing and adult brain in a mouse model of 22q11 deletion syndrome. Mol Cell Neurosci 33(4), 412-428.

Miller, B.J., Buckley, P., Seabolt, W., Mellor, A., Kirkpatrick, B., 2011. Meta-analysis of cytokine alterations in schizophrenia: clinical status and antipsychotic effects. Biol Psychiatry 70(7), 663-671.

Ota, V.K., Belangero, S.I., Gadelha, A., Bellucco, F.T., Christofolini, D.M., Mancini, T.I., Ribeiro-dos-Santos, A.K., Santos, S.E., Mari Jde, J., Bressan, R.A., Melaragno, M.I., Smith Mde, A., 2010. The UFD1L rs5992403 polymorphism is associated with age at onset of schizophrenia. J Psychiatr Res 44(15), 1113-1115.

Ota, V.K., Berberian, A.A., Gadelha, A., Santoro, M.L., Ottoni, G.L., Matsuzaka, C.T., Mari, J.J., Melaragno, M.I., Lara, D.R., Smith, M.A., Belangero, S.I., Bressan, R.A., 2013. Polymorphisms in schizophrenia candidate gene UFD1L may contribute to cognitive deficits. Psychiatry Res 209(1), 110-113.

Pandey, G.N., Rizavi, H.S., Zhang, H., Ren, X., 2018. Abnormal gene and protein expression of inflammatory cytokines in the postmortem brain of schizophrenia patients. Schizophr Res 192, 247-254.

Pollmacher, T., Hinze-Selch, D., Mullington, J., 1996. Effects of clozapine on plasma cytokine and soluble cytokine receptor levels. J Clin Psychopharmacol 16(5), 403-409.

Rampino, A., Walker, R.M., Torrance, H.S., Anderson, S.M., Fazio, L., Di Giorgio, A., Taurisano, P., Gelao, B., Romano, R., Masellis, R., Ursini, G., Caforio, G., Blasi, G., Millar, J.K., Porteous, D.J., Thomson, P.A., Bertolino, A., Evans, K.L., 2014. Expression of DISC1-interactome members correlates with cognitive phenotypes related to schizophrenia. PLoS One 9(6), e99892.

Sachs, N.A., Sawa, A., Holmes, S.E., Ross, C.A., DeLisi, L.E., Margolis, R.L., 2005. A frameshift mutation in Disrupted in Schizophrenia 1 in an American family with schizophrenia and schizoaffective disorder. Mol Psychiatry 10(8), 758-764.

Sanders, A.R., Goring, H.H., Duan, J., Drigalenko, E.I., Moy, W., Freda, J., He, D., Shi, J., Gejman, P.V., 2013. Transcriptome study of differential expression in schizophrenia. Hum Mol Genet 22(24), 5001-5014.

Santarelli, D.M., Beveridge, N.J., Tooney, P.A., Cairns, M.J., 2011. Upregulation of dicer and microRNA expression in the dorsolateral prefrontal cortex Brodmann area 46 in schizophrenia. Biol Psychiatry 69(2), 180-187.

Sarvari, A.K., Vereb, Z., Uray, I.P., Fesus, L., Balajthy, Z., 2014. Atypical antipsychotics induce both proinflammatory and adipogenic gene expression in human adipocytes in vitro. Biochem Biophys Res Commun 450(4), 1383-1389.

Shifman, S., Levit, A., Chen, M.L., Chen, C.H., Bronstein, M., Weizman, A., Yakir, B., Navon, R., Darvasi, A., 2006. A complete genetic association scan of the 22q11 deletion region and

functional evidence reveal an association between DGCR2 and schizophrenia. Hum Genet 120(2), 160-170.

Thiselton, D.L., Vladimirov, V.I., Kuo, P.H., McClay, J., Wormley, B., Fanous, A., O'Neill, F.A., Walsh, D., Van den Oord, E.J., Kendler, K.S., Riley, B.P., 2008. AKT1 is associated with schizophrenia across multiple symptom dimensions in the Irish study of high density schizophrenia families. Biol Psychiatry 63(5), 449-457.

Tkachev, D., Mimmack, M.L., Ryan, M.M., Wayland, M., Freeman, T., Jones, P.B., Starkey, M., Webster, M.J., Yolken, R.H., Bahn, S., 2003. Oligodendrocyte dysfunction in schizophrenia and bipolar disorder. Lancet 362(9386), 798-805.

van Beveren, N.J., Buitendijk, G.H., Swagemakers, S., Krab, L.C., Roder, C., de Haan, L., van der Spek, P., Elgersma, Y., 2012a. Marked reduction of AKT1 expression and deregulation of AKT1-associated pathways in peripheral blood mononuclear cells of schizophrenia patients. PLoS One 7(2), e32618.

van Beveren, N.J., Krab, L.C., Swagemakers, S., Buitendijk, G.H., Boot, E., van der Spek, P., Elgersma, Y., van Amelsvoort, T.A., 2012b. Functional gene-expression analysis shows involvement of schizophrenia-relevant pathways in patients with 22q11 deletion syndrome. PLoS One 7(3), e33473.

Xie, L., Ye, L., Ju, G., Xu, Q., Zhang, X., Liu, S., Shi, J., Yu, Y., Wang, Z., Shen, Y., Wei, J., 2008. A family- and population-based study of the UFD1L gene for schizophrenia. Am J Med Genet B Neuropsychiatr Genet 147B(7), 1076-1079.

Xu, B., Roos, J.L., Dexheimer, P., Boone, B., Plummer, B., Levy, S., Gogos, J.A., Karayiorgou, M., 2011. Exome sequencing supports a de novo mutational paradigm for schizophrenia. Nat Genet 43(9), 864-868.

Xu, B., Roos, J.L., Levy, S., van Rensburg, E.J., Gogos, J.A., Karayiorgou, M., 2008. Strong association of de novo copy number mutations with sporadic schizophrenia. Nat Genet 40(7), 880-885.

Zhang, Z., Convertini, P., Shen, M., Xu, X., Lemoine, F., de la Grange, P., Andres, D.A., Stamm, S., 2013. Valproic acid causes proteasomal degradation of DICER and influences miRNA expression. PLoS One 8(12), e82895.

Zhou, Y., Wang, J., Lu, X., Song, X., Ye, Y., Zhou, J., Ying, B., Wang, L., 2013. Evaluation of six SNPs of MicroRNA machinery genes and risk of schizophrenia. J Mol Neurosci 49(3), 594-599.

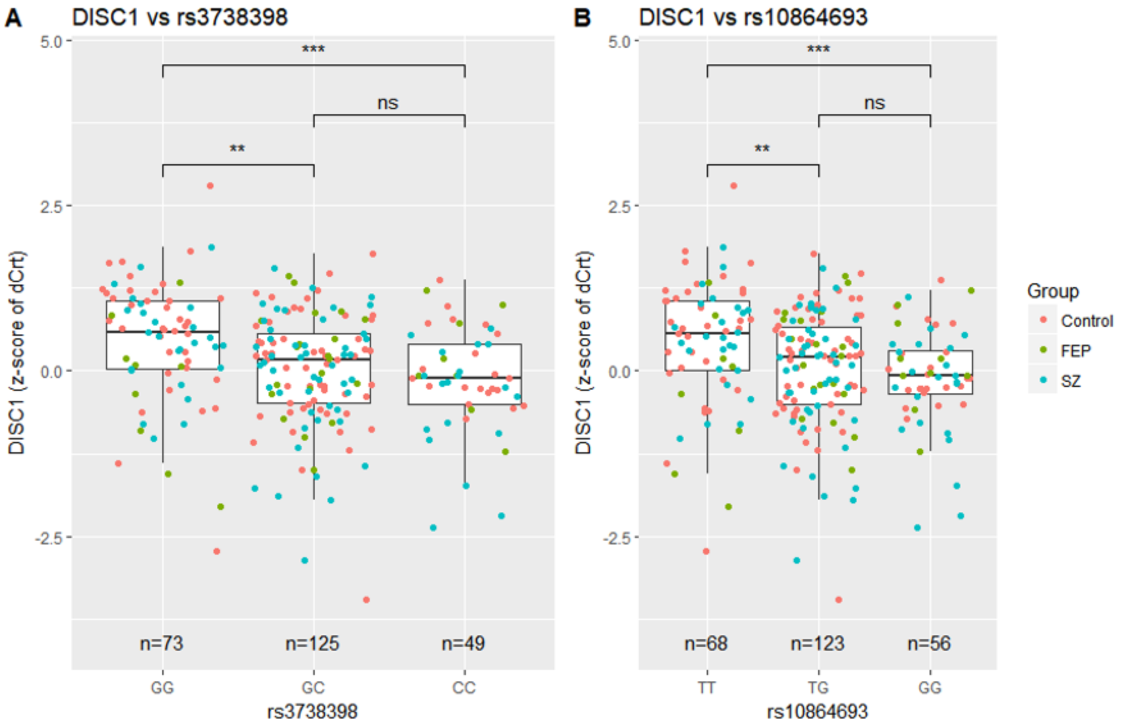

Supplementary Figure S1: Boxplots comparing blood *DISC1* ΔCrt (which is negatively correlated with gene expression) and rs3738398 (A) and rs10864693 (B) genotypes, which are considered as expression Quantitative Trait Loci (eQTL) of *DISC1* in blood by GTEx project. **: post-hoc *p*<0.01; *: post-hoc *p*<0.001; ns: non significant.**

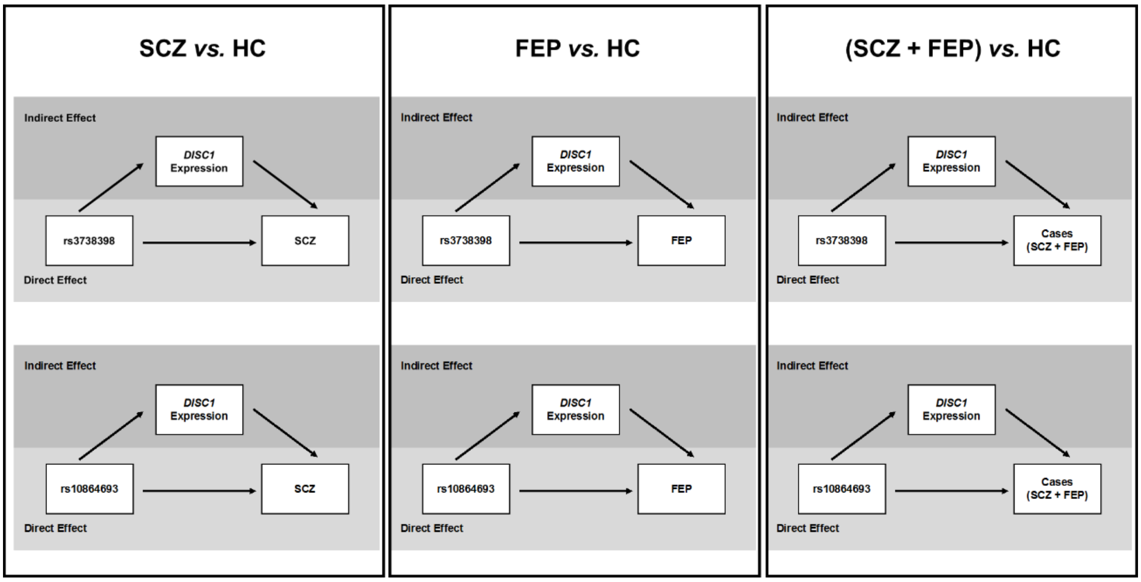

Supplementary Figure S2: Mediation model in which a putative cause (SNP: rs3738398 or rs10864693) is related to a presumed effect (disorder: schizophrenia (SCZ), First-episode psychosis (FEP), or SCZ + FEP) via an intermediate variable (*DISC1* expression).

Supplementary Notes

For 69 controls, 60 FEP and 124 CSZ, we had data on smoking. Thus, we verified if any of the target genes was associated with smoking status, using GLM and inserting age as covariate and smoking and sex as fixed factors, and none was significant ($p>0.05$).

Also, for 47 controls and 48 FEP we have predicted blood cell count based on methylation analysis from Infinium Human Methylation 450K BeadChip. Thus, we performed a GLM analysis, considering each gene expression as dependent variable and age and cell count as covariates and sex as fixed factor, and only *DICER1* and *NDEL1* were significantly associated with specific cell types, as shown in the table below. *DICER1* was not a significant gene in our analyses, and when we inserted Bcell Mono and Gran cells as covariates in the GLM, no significant result was found. In contrast, when we included Bcell as covariate in the GLM, *NDEL1* remained associated with groups (adjusted $p = 8.31\times10^{-4}$). However, as we only have this cell count data for some FEP and controls, we cannot extrapolate to other comparisons, and we did not include this analysis in our main draft.

Table: Adjusted p-values (for 12 comparisons) for each blood cell using general linear model.

Gene	**CD8T**	**CD4T**	**NK**	**Bcell**	**Mono**	**Gran**
AKT1	1.000	1.000	1.000	0.708	1.000	1.000
COMT	1.000	1.000	1.000	1.000	1.000	1.000
DGCR2	1.000	1.000	1.000	1.000	1.000	1.000
DGCR8	1.000	1.000	1.000	1.000	1.000	1.000
DICER1	0.084	0.12	0.096	0.012	0.036	0.048
DISC1	1.000	1.000	1.000	1.000	1.000	1.000
DROSHA	1.000	1.000	1.000	1.000	1.000	1.000
MBP	1.000	0.672	1.000	0.876	0.144	0.576
NDEL1	0.18	0.24	0.168	0.024	0.168	0.036
PAFAH1B1	1.000	1.000	1.000	0.3	1.000	1.000
TNF	1.000	1.000	1.000	1.000	1.000	1.000
UFD1	1.000	1.000	1.000	1.000	1.000	1.000
